# Predicting the Common Carotid Artery Bifurcation: A Comparative Assessment of Landmark-Based Methods

**DOI:** 10.7759/cureus.87552

**Published:** 2025-07-08

**Authors:** Sampath Kumar, Sebastian T Kiehn, Lena M Duenas, Steven N Popoff, Nicole L Griffin

**Affiliations:** 1 Department of Biomedical Education and Data Science, Lewis Katz School of Medicine at Temple University, Philadelphia, USA

**Keywords:** anatomical landmarks, carotid bifurcation, carotid endarterectomy, predictive model, vascular anatomy

## Abstract

Introduction: The common carotid artery (CCA) bifurcation is a critical landmark in vascular surgery. There is a pressing need for a non-invasive, predictive method to accurately locate the CCA bifurcation. Using easily palpable anatomical landmarks, including the medial border of the clavicle (MBC), jugular notch (JN), and mastoid process (MP), we developed two new models for locating the CCA bifurcation and assessed their predictive reliability relative to a previously published method.

Materials and methods: Formalin-fixed human donors (n=30) were selected based on their suitability for comprehensive anterior neck dissections. Prior to dissection, straight-line measurements were obtained from the JN/MBC to the MP for each donor. Following neck dissections, secondary straight-line measurements were made from the JN/MBC to the MP and the CCA bifurcation. The location of the CCA bifurcation was predicted using three models. Model I involved multiplying the MBC-MP distance by 0.65/0.74 (right/left side) using a method described in a previously published study; Model II involved multiplying the MBC-MP distance by 0.59/0.57; and Model III involved multiplying the JN-MP distance by 0.585/0.58. The location of the CCA bifurcation as predicted by these models using pre- and post-dissection measurements was then compared with the actual location of the bifurcation to assess the external and internal validity, respectively, of each model.

Results: The CCA bifurcation was, on average, located 4.23 mm higher on the right side than on the left side of the body when measuring from the MBC (p=0.026). However, after normalization for neck length, no significant difference was observed. Although bifurcation location based on Model I using pre-dissection measurements significantly deviated from actual measurements for both the left (p<0.0001) and right (p=0.002) sides, there were no significant differences between actual and predicted measurements for Model II or Model III. Similarly, using post-dissection measurements, Model I showed significant deviations from actual measurements on both the left (p < 0.0001) and right (p < 0.0001) sides, whereas there was no significant difference between actual and predicted measurements for Models II and III.

Conclusion: We propose two new models for predicting the location of the CCA bifurcation using palpable landmarks: the MBC (Model II) and the JN (Model III). We noted that the latter method may prove to be more practical in the clinical setting. These non-invasive models could improve preoperative planning, surgical outcomes, and patient safety for carotid stenosis and stroke prevention. Future directions for this research include validating our models in other donor samples and clinical scenarios.

## Introduction

Carotid endarterectomy is the primary surgical procedure for treating severe carotid stenosis [1-3]. This operation involves making an incision in the neck to access the common carotid artery (CCA), removing the atherosclerotic plaque, and repairing the artery to restore blood flow [1,3]. Accurate identification of the CCA bifurcation (CCAB) is crucial for incision placement [1,4]. While anatomy textbooks typically describe the bifurcation as occurring at the superior border of the thyroid cartilage [5,6], the research literature has documented variation within and across individuals. The vertical position of the carotid bifurcation has been reported to range from as high as the lower third of the C2 vertebral body to as low as the upper third of C6, with the most common location at the lower level of C3 [7-10]. This variability has contributed to the difficulty in predicting the location preoperatively without the use of diagnostic imaging. Although a model was recently proposed [11], there is currently no validated method for predicting the bifurcation based on easily palpable landmarks [4,12]. The purpose of the current study is to test and validate the proposed model, as well as to develop an alternative model for accurately predicting the location of the CCA prior to surgery when diagnostic imaging is not available. This article was previously presented as a meeting poster at the 2024 AAA Anatomy Connected Meeting on March 24, 2024.

## Materials and methods

Formalin-fixed donors (n=30; 15 males; 15 females) were selected from the Lewis Katz School of Medicine at Temple University's Gross Anatomy Laboratory based on their suitability for anterior neck dissection. The donors ranged in age from 58 to 105 years, with an average of 85 years. No donors showed signs of trauma or other visible abnormalities. The medial border of the clavicle (MBC), mastoid process (MP), and jugular notch (JN) were identified prior to dissection by palpation and marked for measurement. A single, experienced anatomist was involved in the palpation and identification of each identified landmark. The bony landmark of the MBC was identified using the methods described by Griepp et al. [11]. The MP was located by palpating for the tip of the process posteroinferior to the opening of the external acoustic meatus. The JN was determined by palpating for the manubrium and the sternocleidomastoid muscle on the left and right sides. Straight-line measurements from the MBC to the MP (n=15) and from JN to the MP (n=19) were taken bilaterally in donors with palpable landmarks. The sample size for MBC to MP and JN to MP measurements was not uniform due to the inability to reliably palpate these anatomical landmarks in several donors with a large body mass index (BMI), which limited inclusion in these groups. All measurements were made using a Neiko 01409A electronic digital caliper (Conegliano, Italy) with a resolution of up to a hundredth of a millimeter. To minimize measurement bias arising from variable neck curvature or soft tissue deformation, all cadavers were positioned supine with their necks extended as far as possible in a standardized manner prior to each measurement, ensuring consistent alignment of anatomical landmarks across donors.

**Figure 1:**
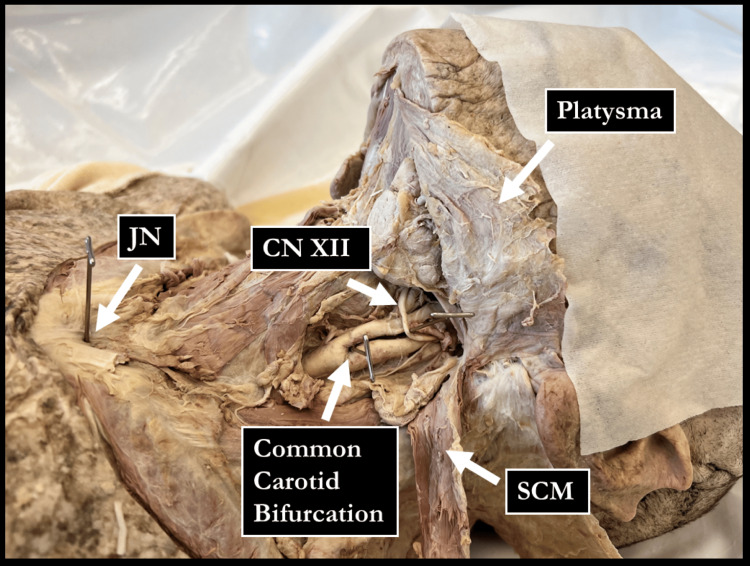
Example of an anterior neck dissection approach used in this study JN: jugular notch; CNXII: hypoglossal nerve; SCM: sternocleidomastoid Image Credits: Sampath Kumar

**Figure 2:**
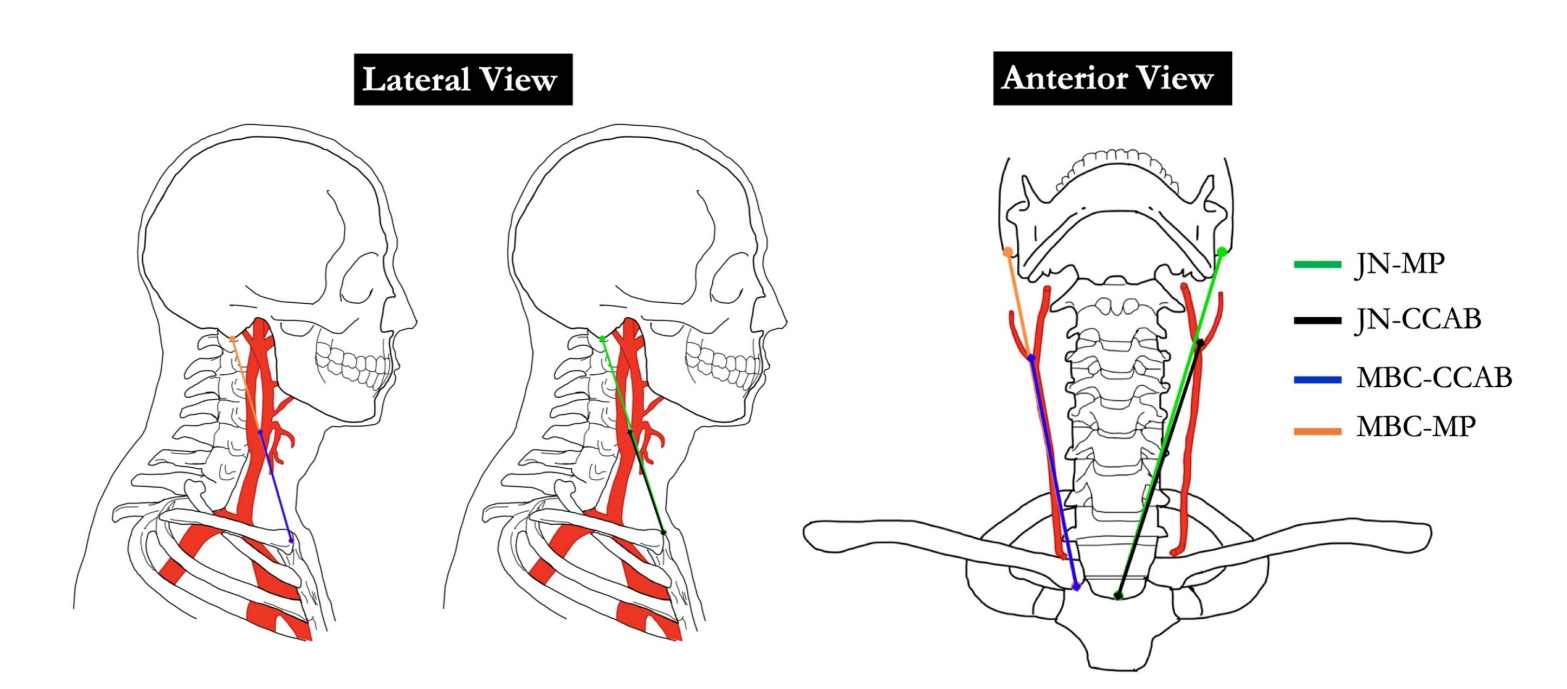
Anterior and lateral views of the landmarks used for distance measurements JN: jugular notch; MP: mastoid process; CCAB: common carotid artery bifurcation; MBC: medial border of the clavicle Image Credits: Sampath Kumar

Following these initial measurements, a dissection of the anterior neck was performed. After skin removal, the platysma and both heads of the SCM were reflected superiorly for identification of the CCA. Care was taken to clearly delineate the CCAB, external carotid artery (ECA), and internal carotid artery (ICA) and to retain the relationship between the CCAB and the hypoglossal nerve (CN XII) (Figure 1). Additionally, the JN and the sternoclavicular junction were exposed. Following dissection, straight-line measurements were taken on both sides of each donor. The following distances were measured: (a) MBC to MP, (b) MBC to CCAB, (c) JN to MP, and (d) JN to CCAB (Figure 2).

Model I was based on the quantitative prediction method described by Griepp et al. [11]. This model used the distance between the MBC and the MP to predict the location of the CCAB. According to Griepp et al. [11], the bifurcation height can be estimated by multiplying the MBC-MP distance by 0.65 for the right side and 0.74 for the left side. This model was included as a baseline for comparison with Models II and III developed in our study. Model II was calculated using the same method as described in Griepp et al. [11] by dividing the MBC-CCAB distance by the MBC-MP distance from our donors. Model III was calculated by dividing the JN-CCAB distance by the JN-MB distance from our donors. The measured CCAB location for each donor was then compared to the calculated bifurcation location determined by Models I, II, and III (see below). Data were collected in Microsoft Excel (Microsoft Corporation, Redmond, Washington) and then transferred into GraphPad Prism, Version 10.0.0 (GraphPad Software, San Diego, California) for statistical analysis. Paired t-tests were run for bilateral comparisons, and a p-value of less than 0.05 was considered significant.

\begin{align*} \text{Model I\char58} \quad & \text{MBC to CCAB distance} = (\text{MBC to MP distance}) \times \begin{cases} 0.65 & \text{for Left } (L) \\ 0.74 & \text{for Right } (R) \end{cases} \end{align*}

\begin{align*} \text{Model II\char58} \quad & \text{MBC to CCAB distance} = (\text{MBC to MP distance}) \times \begin{cases} 0.59 & \text{for Left } (L) \\ 0.57 & \text{for Right } (R) \end{cases} \end{align*}

\begin{align*} \text{Model III\char58} \quad & \text{MBC to CCAB distance} = (\text{JN to MP distance}) \times \begin{cases} 0.585 & \text{for Left } (L) \\ 0.58 & \text{for Right } (R) \end{cases} \end{align*}

## Results

All measurements are reported in Table 1. Bilateral symmetry for each measurement was analyzed. The mean distance from the MBC to the CCAB was significantly asymmetric (p = 0.026), with the right side being 4.23 mm higher on average. The distance from the MBC to the MP and from the JN to the CCAB did not show statistically significant differences between the left and right measurements (Table 2).

**Table 1:** Summary measurements JN: jugular notch; MP: mastoid process; CCAB: common carotid artery bifurcation; MBC: medial border of the clavicle; MP: mastoid process; SEM: standard error of the mean; SD: standard deviation

Distance in mm (n=60)	Mean	SEM	Median	SD	Variance	Range (Min–Max)
MBC to MP	163.30	2.15	164.90	16.66	277.56	78.23 (127.90–206.20)
MBC to CCAB	95.12	1.77	94.86	13.74	188.79	62.49 (65.57–128.10)
JN to MP	187.60	2.14	190.70	16.56	274.23	71.44 (150.70–222.10)
JN to CCAB	108.90	1.68	108.70	12.97	168.22	62.92 (80.87–143.80)

**Table 2:** Summary of left vs. right measurements Statistical significance was defined as p < 0.05 and is denoted as follows: *p < 0.05; **p < 0.001; ***p < 0.0001 JN: jugular notch; MP: mastoid process; CCAB: common carotid artery bifurcation; MBC: medial border of the clavicle; SE: standard error; SD: standard deviation; CI: confidence interval

Distance in mm (n=30)	Left Mean ± SD	Right Mean ± SD	p-value	Mean Difference (R-L)	SE Difference	95% CI for Mean Difference	
						Lower	Upper
MBC to MP	161.80 ± 19.12	164.80 ± 13.94	0.202	2.95	2.27	-1.68	7.59
MBC to CCAB	93.00 ± 14.85	97.23 ± 12.44	0.026*	4.23	1.80	.56	7.91
JN to MP	186.80 ± 17.34	188.40 ± 16.01	0.346	1.58	1.65	-1.79	4.94
JN to CCAB	108.00 ± 13.58	109.80 ± 12.50	0.216	1.87	1.48	-1.15	4.90

Neck length was normalized by dividing the MBC-CCAB and JN-CCAB distances by the MBC-MP and JN-MP distances, respectively. This was done to correct for any potential effect that neck length may have on our measurements. The mean normalized ratio for the distance from the MBC to the CCAB was 0.573 on the left side and 0.590 on the right side. No significant difference was observed between the left and right side mean normalized ratio (p = 0.057). Similarly, the normalized ratio for the distance from the JN to the CCAB showed no significant difference between the left and right sides (Table 3).

**Table 3:** Summary of left vs. right normalized ratios Statistical significance was defined as p < 0.05 and is denoted as follows: *p < 0.05; **p < 0.001; ***p < 0.0001 JN: jugular notch; CCAB: common carotid artery bifurcation; MBC: medial border of the clavicle; SE: standard error; SD: standard deviation; CI: confidence interval

Distance in mm (n=30)	Left Mean ± SD	Right Mean ± SD	p-value	Mean Difference (R-L)	SE Difference	95% CI for Mean Difference	
						Lower	Upper
MBC to CCAB	0.573 ± 0.046	0.590 ± 0.052	0.057	0.016	0.008	-0.001	0.033
JN to CCAB	0.580 ± 0.067	0.585 ± 0.064	0.452	0.005	0.007	-0.009	0.019

The internal validity of the three predictive models was assessed by comparing the predicted CCAB locations to the actual post-dissection measurements (Table 4). Model I showed a statistically significant predicted mean deviation from actual measurements with a mean difference of 9.88 mm (p < 0.0001) on the right side and 26.75 mm on the left side (p < 0.0001). Model II did not show a statistically significant predicted mean deviation from actual measurements, with a mean difference of -0.01 mm (p = 0.995) on the right side and -0.033 mm on the left side (p = 0.985). Model III also did not show a statistically significant predicted mean deviation from actual measurements, with a mean difference of 0.346 mm (p = 0.88) on the right side and 0.361 mm on the left side (p = 0.88).

**Table 4:** Post-dissection/internal validity of the predicted bifurcation Statistical significance was defined as p < 0.05 and is denoted as follows: *p < 0.05; **p < 0.001; ***p < 0.0001 JN: jugular notch; MP: mastoid process; CCAB: common carotid artery bifurcation; MBC: medial border of the clavicle; SE: standard error; SD: standard deviation; CI: confidence interval

Model	Side	Predicted Mean From Post-dissection Measurements	Actual Mean Measured Post-dissection	p-value	Mean Difference	SE Difference	95% CI for Mean Difference		
							Lower	Upper	
Model I (Published method) (n=30)	Right: MBC to CCAB	107.10 ± 9.06	97.23 ± 12.44	<0.0001***	9.88	1.52	6.78		12.98
	Left: MBC to CCAB	119.80 ± 14.15	93.00 ± 14.85	<0.0001***	26.75	1.37	23.95		29.56
Model II (MBC Novel method) (n=30)	Right: MBC to CCAB	97.22 ± 8.22	97.23 ± 12.44	0.995	-.010	1.53	-3.13		3.11
	Left: MBC to CCAB	92.97 ± 10.28	93.00 ± 14.85	0.985	-.033	1.70	-3.51		3.44
Model III (JN Novel method) (n=30)	Right: JN to CCAB	110.20 ± 9.37	109.80 ± 12.50	0.880	.346	2.26	-4.28		4.97
	Left: JN to CCAB	108.30 ± 10.05	108.00 ± 13.58	0.880	.361	2.37	-4.48		5.20

The external validity of the three predictive models was assessed by comparing the predicted CCAB locations to the actual pre-dissection measurements. Model I showed a statistically significant predicted mean deviation from actual measurements with a mean difference of 10.66 mm (p = 0.002) on the right side and 33.24 mm on the left side (p < 0.0001). Model II did not show a statistically significant predicted mean deviation from actual measurements, with a mean difference of 0.584 mm (p = 0.841) on the right side and 4.64 mm on the left side (p = 0.236). Model III also did not show a statistically significant predicted mean deviation from actual measurements, with a mean difference of 2.38 mm (p = 0.482) on the right side and 2.51 mm on the left side (p = 0.465) (Table 5).

**Table 5:** Pre-dissection/external validity of the predicted bifurcation Statistical significance was defined as p < 0.05 and is denoted as follows: *p < 0.05; **p < 0.001; ***p < 0.0001 JN: jugular notch; MP: mastoid process; CCAB: common carotid artery bifurcation; MBC: medial border of the clavicle; SE: standard error; SD: standard deviation; CI: confidence interval

Model	Side	Predicted Mean From Post-dissection Measurements	Actual Mean Measured Post-dissection	p-value	Mean Difference	SE Difference	95% CI for Mean Difference		
							Lower	Upper	
Model I (Published method) (n=15)	Right: MBC to CCAB	109.20 ± 8.09	98.53 ± 12.90	.002*	10.66	2.88	4.50		16.83
	Left: MBC to CCAB	124.50 ± 13.83	91.26 ± 14.17	<0.0001***	33.24	4.15	24.35		42.13
Model II (MBC Novel method) (n=15)	Right: MBC to CCAB	99.11 ± 7.34	98.53 ± 12.90	0.841	.584	2.86	-5.55		6.71
	Left: MBC to CCAB	95.90 ± 10.65	91.26 ± 14.17	0.236	4.64	3.75	-3.40		12.68
Model III (JN Novel method) (n=19)	Right: JN to CCAB	110.70 ± 9.34	108.40 ± 12.57	0.482	2.38	3.32	-4.59		9.35
	Left: JN to CCAB	108.40 ± 9.76	105.90 ± 12.33	0.465	2.51	3.36	-4.54		9.56

**Figure 3:**
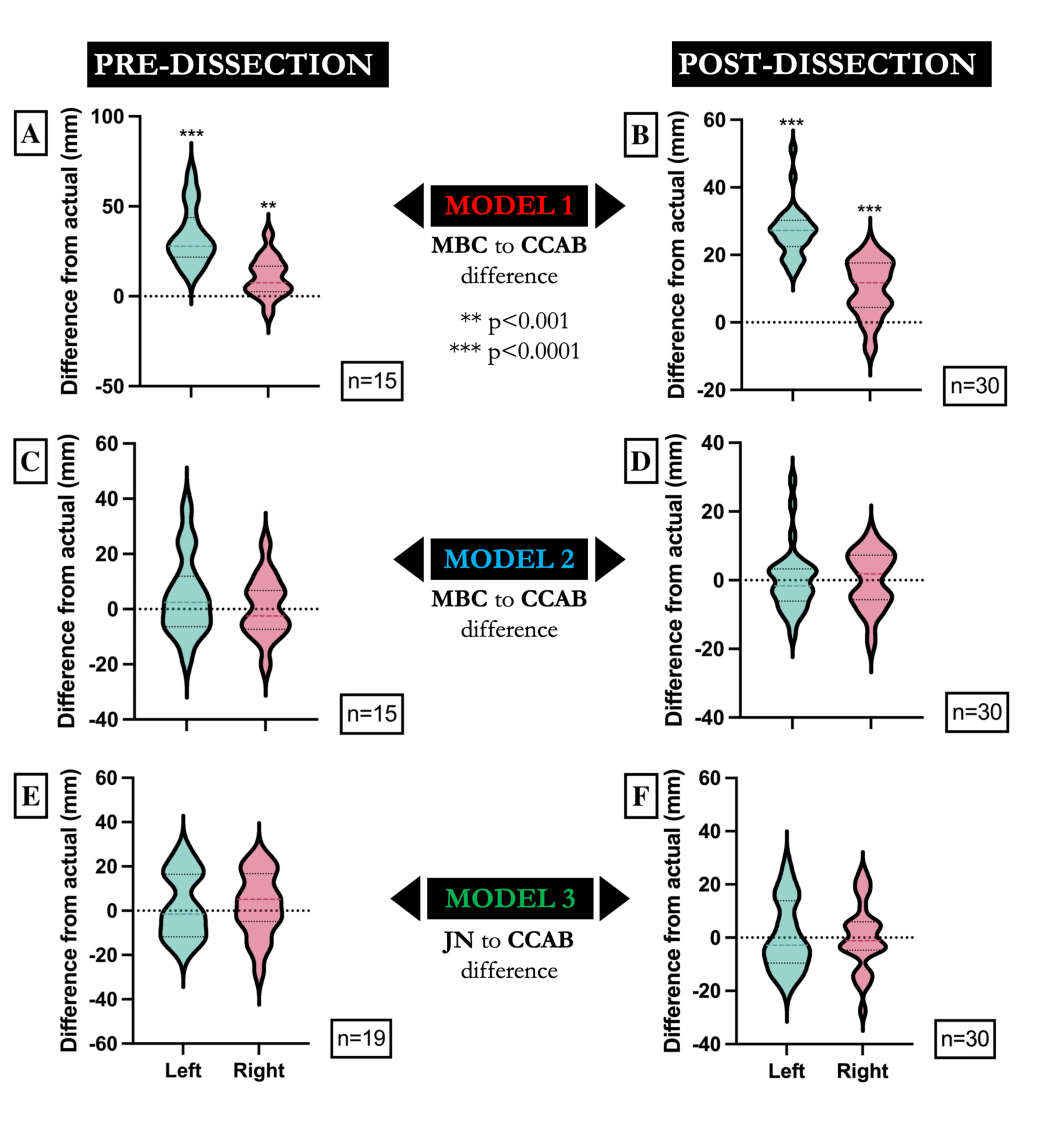
Mandolin plots for each model (I–III) depicting the distance between the predicted bifurcation location and actual bifurcation location in millimeters (y-axis) for the left and right sides of the donor sample (x-axis) taken before and after dissection Model 1 (MBC to CCAB difference) shows a significant difference in the estimation of the carotid bifurcation location, both pre-dissection (A) and post-dissection (B). Model 2 (MBC to CCAB difference) demonstrates no significant difference from actual measurements in both pre-dissection (C) and post-dissection (D) conditions. Model 3 (JN to CCAB difference) demonstrates no significant difference from actual measurements for both pre-dissection (E) and post-dissection (F) conditions. The sample sizes for each group are indicated within each panel. Statistical significance is denoted as follows: **p < 0.001, ***p < 0.0001. MBC: medial border of the clavicle; CCAB: common carotid artery bifurcation; JN: jugular notch

Figure 3 illustrates the distribution of prediction errors for each model, comparing the difference from actual CCAB measurements for both pre- and post-dissection data. Model I demonstrates a significant positive deviation from actual measurements on both sides (p < 0.001), whereas Models II and III show distributions centered around zero, indicating no significant difference between the predicted and actual values. These findings visually corroborate the statistical results presented in Tables 4, 5.

The accuracy of the models was also assessed against an acceptable error of 1.5 cm [11]. Models II and III had a higher number of predictions within 1.5 cm of the expected height compared to Model I for both pre- and post-dissection (Table 6).

**Table 6:** Comparison of predictive methods with respect to acceptable error

Model	No. of predictions within 1.5 cm of expected height/total no. of predictions			
	Pre-dissection		Post-dissection	
	Number	Percentage	Number	Percentage
Model I	10/30	33.33	21/60	35.00
Model II	24/30	80.00	55/60	91.67
Model III	30/38	78.95	53/60	88.33

## Discussion

This study aimed to develop and validate non-invasive models for predicting the location of the CCAB using easily palpable anatomical landmarks. We first tested a previously defined method [11] for estimating the distance between the MBC and CCAB, using the distance between the MBC and MP. We then developed a novel method by using the distance between the JN-MBC.

Bilateral measurements showed a significant asymmetry in the mean distance from the MBC to the CCAB, with the right side being higher. However, there were no significant differences for the other measured distances or for the normalized ratios, indicating overall bilateral similarity except for the MBC-CCAB measurement.

Models II and III were calibrated using the collected data, and when applied to their respective pre-dissection measurements of the MBC-MP or JN-MP, they accurately predicted bifurcation within acceptable surgical error 80% and 78.95% of the time. These two models demonstrated significantly higher predictive accuracy than Model I, which was only able to predict the bifurcation location within an acceptable surgical error in 33.33% of cases. This accuracy was also demonstrated by comparing the mean estimated and measured distances between the MBC-MP in Models I and II and the distance between the JN and MP in Model III. Models II and III showed no significant difference between the predicted and measured mean distance (Table 4). In comparison, Model I showed a statistically significant difference between the predicted and measured mean distance (Table 4).

Models I and II use the same fundamental equation but use different scaling factors to convert the MBC-MP distance to the MBC-CCAB distance. The difference in scaling factor is due to the fact that for Model II, the scaling factor was calculated using data from this project, whereas the scaling factor for Model I was calculated from data by Griepp et al. [11]. One of the major differences between the two models was the scaling factors in Griepp et al., which showed significant variation in the left and right variance, with a right scaling factor of 0.65 and a left scaling factor of 0.74. This variation was not observed in Model II, which had a right-side scaling factor of 0.59 and a left-side scaling factor of 0.57, indicating only a slight increase in the right-sided scaling factor compared to the left. Data indicate that there can be significant variation in the left-to-right bifurcation height within patients; however, there is no consensus that the bifurcation height is skewed, with the left side being higher [4,13].

The non-invasive models developed in this study have significant clinical implications. In circumstances when ultrasound is not available, accurate prediction of the CCAB location can improve preoperative planning and surgical outcomes in procedures such as carotid endarterectomy. The use of easily palpable landmarks, such as the JN and MBC, can enhance patient safety by reducing the risk of intraoperative complications [14,15]. Locating the JN was found to be more practical and easier compared to the MBC, suggesting that Model III may be more suitable for clinical applications.

Study limitations

Despite the promising results demonstrated by Models II and III, several limitations should be considered when interpreting the findings of this study. First, the sample size was relatively small and comprised exclusively of cadaveric donors from a single institution, which may limit the generalizability of the results to broader or more diverse populations, including living patients. The advanced age and unknown medical histories of some donors may have also introduced unrecognized anatomical variability. Additionally, the advanced age of the donor population (average age: 85 years) is associated with increased vessel tortuosity, muscular atrophy, and other age-related anatomical changes [16,17]. These factors, in combination with well-known limitations of cadaveric studies such as tissue shrinkage and fixation distortion, may further impact the accuracy of anatomical measurements [18]. As a result, the findings of this study may not be generalizable to younger individuals or to live subjects, where vascular and soft tissue characteristics can differ substantially. Quantification of post-fixation changes and age-related anatomical variation would be desirable in future studies to provide stronger evidence and improve the applicability of these predictive models.

Second, both predictive models were developed and validated using the same dataset, increasing the possibility of overfitting and potentially overestimating their predictive accuracy when applied to external populations. A separate validation may be carried out in the near future for Models II and III to address this limitation and better assess their generalizability.

Third, while the study relied on easily palpable anatomical landmarks, inter-rater variability in landmark identification and measurement could have affected reproducibility and accuracy in clinical settings. Furthermore, straight-line measurements between landmarks did not account for soft tissue displacement or patient positioning differences that may occur during live surgery. Although care was taken to standardize measurement techniques, no method was employed to fully eliminate the influence of individual neck curvature or soft tissue deformation, which may introduce variability and potential bias in the straight-line measurements across different specimens. Finally, this study did not assess the performance of these models in living subjects or in the presence of pathological conditions such as tumors or vascular anomalies, which could further impact the accuracy of landmark-based predictions.

## Conclusions

In conclusion, we propose two new models for predicting the CCAB location using palpable landmarks. Model II (MBC-based) and Model III (JN-based) demonstrated high predictive accuracy and practicality, with the JN-based model being particularly suitable for clinical use. These models have the potential to significantly enhance surgical planning and patient outcomes in vascular surgery. The results are consistent with previous findings that emphasize the importance of anatomical landmarks in predicting the CCAB and their clinical relevance in surgical procedures. Future research should focus on validating these models in larger, more diverse samples and in clinical scenarios to confirm their utility and reliability.
